# TiN Paper for Ultrafast-Charging Supercapacitors

**DOI:** 10.1007/s40820-019-0340-7

**Published:** 2019-12-10

**Authors:** Bin Yao, Mingyang Li, Jing Zhang, Lei Zhang, Yu Song, Wang Xiao, Andrea Cruz, Yexiang Tong, Yat Li

**Affiliations:** 1 Department of Chemistry and Biochemistry, University of California, Santa Cruz, Santa Cruz, CA 95064 USA; 2 KLGHEI of Environment and Energy Chemistry, MOE of the Key Laboratory of Bioinorganic and Synthetic Chemistry, School of Chemistry and Chemical Engineering, Sun Yat-Sen University, Guangzhou, 510275 People’s Republic of China; 3 State Key Laboratory of Advanced Technology for Materials Synthesis and Processing, Wuhan University of Technology, Wuhan, 430070 People’s Republic of China

**Keywords:** Ultrafast charging, Wide voltage window, TiN, Paper-like electrode, Supercapacitors

## Abstract

Ultrafast-charging energy storage devices are attractive for powering personal electronics and electric vehicles. Most ultrafast-charging devices are made of carbonaceous materials such as chemically converted graphene and carbon nanotubes. Yet, their relatively low electrical conductivity may restrict their performance at ultrahigh charging rate. Here, we report the fabrication of a porous titanium nitride (TiN) paper as an alternative electrode material for ultrafast-charging devices. The TiN paper shows an excellent conductivity of 3.67 × 10^4^ S m^−1^, which is considerably higher than most carbon-based electrodes. The paper-like structure also contains a combination of large pores between interconnected nanobelts and mesopores within the nanobelts. This unique electrode enables fast charging by simultaneously providing efficient ion diffusion and electron transport. The supercapacitors (SCs) made of TiN paper enable charging/discharging at an ultrahigh scan rate of 100 V s^−1^ in a wide voltage window of 1.5 V in Na_2_SO_4_ neutral electrolyte. It has an outstanding response time with a characteristic time constant of 4 ms. Significantly, the TiN paper-based SCs also show zero capacitance loss after 200,000 cycles, which is much better than the stability performance reported for other metal nitride SCs. Furthermore, the device shows great promise in scalability. The filtration method enables good control of the thickness and mass loading of TiN electrodes and devices.

**Electronic supplementary material:**

The online version of this article (10.1007/s40820-019-0340-7) contains supplementary material, which is available to authorized users.

## Highlights

- The superior conductivity and unique porous electrode structure in the TiN paper enable fast charging by simultaneously providing efficient ion diffusion and electron transport.
- The TiN paper-based supercapacitors exhibit charging/discharging at an ultrahigh scan rate of 100 V s^−1^ in a wide voltage window of 1.5 V in Na_2_SO_4_ neutral electrolyte and show zero capacitance loss after 200,000 cycles.

**Electronic supplementary material:**

The online version of this article (10.1007/s40820-019-0340-7) contains supplementary material, which is available to authorized users.

## Graphical abstract

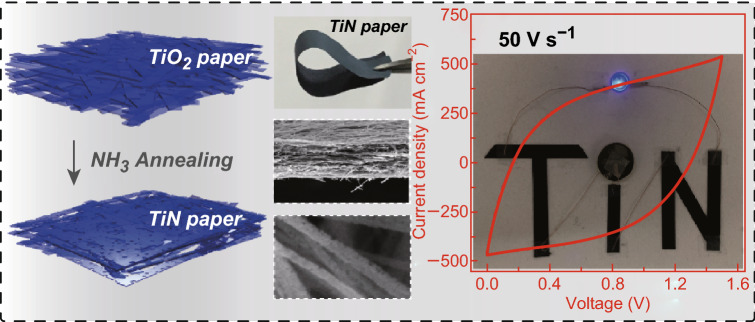

## Introduction

The emerging advances in portable and wearable electronics urge the rapid development of fast charging and discharging energy storage devices [1–6]. Supercapacitors (SCs), renowned for their high charging rate and long lifespan, have received great attention in the past decades [7, 8]. Currently, most SCs are operated at a charging speed of 2–100 mV s^−1^, which corresponds to the charging time from tens of seconds to tens of minutes [9, 10]. Further increasing the charging speed usually results in an inferior performance and deteriorated material structure [11]. Ultrafast-charging SCs with charging rate > 10 V s^−1^ would significantly shorten the charging time and meet the requirements for high-rate energy storage devices [12–14]. Ultrafast-charging SCs are mainly fabricated by carbonaceous materials, such as activated carbon, graphene, and carbon nanotubes (CNTs) [15–18]. Yet, the relatively low electrical conductivity of activated carbon (~ 1 to 100 S m^−1^), chemically converted graphene (~ 500 to 2000 S m^−1^), and CNTs (~ 1 × 10^4^ S m^−1^) restricts their performances at ultrahigh charging rates [19, 20]. Therefore, exploration of new materials with superior conductivity for ultrafast-charging SCs is highly desirable.

Transition metal nitrides have received increasing attention for energy storage devices due to their excellent electrical conductivity and high capacities/capacitances [21, 22]. Among them, titanium nitride (TiN) stands out as one of the most promising material for SCs because of its outstanding conductivity (4 × 10^5^ ~ 5.55 × 10^6^ S m^−1^) and mechanical stability [22, 23]. However, in most cases, TiN nanostructures were supported on a substrate, which limits the gravimetric capacitances of TiN electrodes when it is normalized with the mass of entire electrode. Besides, most TiN-based electrodes suffered from severe capacitance loss in aqueous electrolytes, especially in the acidic and alkaline medium [24, 25]. Hasegawa et al. [25] showed that the neutral electrolyte can help to alleviate the cycling stability of transition metal nitrides. Here, we report the fabrication of a freestanding, flexible and porous TiN paper electrode for ultrafast-charging SCs. Electrical measurements showed that a single TiN nanobelt and a piece of TiN paper achieved excellent conductivities of 4.5 × 10^5^ and 3.67 × 10^4^ S m^−1^, respectively. The unique combination of high conductivity and pore structure of the TiN paper warrants rapid electron transport and ion diffusion that are required for ultrafast charging. The SC device also shows remarkable stability, which is uncommon for metal nitride materials.

## Materials and Methods

### Synthesis of Ultralong TiO_2_ Nanobelts

P25 powder (0.1 g) was mixed with 20 mL 10 mol L^−1^ NaOH aqueous solution. The mixture was transferred to a Teflon-lined autoclave and heated at 200 °C for 48 h. The autoclave was cooled down at room temperature. The solid product, sodium titanate (Na_2_Ti_3_O_7_) nanobelts, in the solution was collected by vacuum filtration and washed with deionized water. The sodium titanate was then re-dispersed into 0.1 mol L^−1^ HCl aqueous solution and let it stay for 24 h to form hydrogen titanate (H_2_Ti_3_O_7_) nanobelts through ion exchange reaction. Finally, TiO_2_ nanobelts were obtained by annealing the H_2_Ti_3_O_7_ nanobelts at 500 °C in air for 1 h.

### Preparation of TiO_2_ and TiN Papers

TiO_2_ and TiN paper were prepared by vacuum filtration of the ultralong H_2_Ti_3_O_7_ nanobelts followed by annealing process. First, the H_2_Ti_3_O_7_ nanobelts were re-dispersed into 100 mL deionized water and stirred for 0.5 h to make a uniform suspension. Then, the H_2_Ti_3_O_7_ nanobelt suspension solution was poured into the vacuum filtration system to get a H_2_Ti_3_O_7_ paper. The H_2_Ti_3_O_7_ paper with the filter paper was put in an electric oven at 70 °C for 20 min until they get dry. The H_2_Ti_3_O_7_ paper can be easily peeled off from the filter paper afterward. The mass loading of paper electrode can be readily adjusted by changing the amount of H_2_Ti_3_O_7_ nanobelt suspension in the solution for the filtration.

TiO_2_ paper was obtained by annealing the H_2_Ti_3_O_7_ paper at 500 °C in air for 1 h. TiN papers were obtained by annealing the TiO_2_ paper in ammonia environment at 800 °C for 1 h. The conventional TiN pellet electrodes as a control sample were fabricated by mixing the TiN nanobelts, carbon black, and PTFE in a ratio of 8:1:1 followed by rolling the mixture into thin films.

### Materials Characterization

The X-ray diffraction (XRD) patterns were collected on a powder X-ray diffractometer (Rigaku Americas Miniflex Plus) with 2θ angle from 30° to 70° under a step size of 0.01° at a rate of 1° min^−1^. The morphology of nanobelts was investigated by a field emission scanning electron microscopy (SEM, FEI Quanta 3D FEG dual beam) and transmission electron microscopy (TEM, JEM, 2010-HR). X-ray photoelectron spectroscopy (XPS, ESCALAB 250) was used to analyze the chemical composition of samples. Textural properties were examined by Brunauer–Emmett–Teller and Barrett–Joyner–Halenda methods using an ASAP 2020 surface area analyzer (Micromeritics Instrument) via nitrogen porosimetry. The areal mass of the electrodes was measured based on 4 cm^2^ TiN papers on an analytical balance (Citizen CX265) with a resolution of 0.01 mg. The thickness of the electrodes was measured using a micrometer caliper (NSCING) with a resolution of 0.001 mm. The single TiO_2_ and TiN nanobelt devices were fabricated via a focused ion beam (FIB, Quanta 3D FEG) with Pt as the contact electrode. The electrical measurement was carried out using an Agilent 2400 instrument.

The electrochemical measurements were conducted using electrochemical workstation (CHI 660D and EC-Lab SP-300). For three-electrode measurements, a piece of 0.2 cm^2^ TiN paper was used as working electrode. Ag/AgCl (CHI, USA) and YP-50 activated carbon (Kuraray Chemical, Japan) were used as the reference electrode and counter electrode, respectively. The measurements were carried out in different aqueous electrolytes, including 0.5 M Na_2_SO_4_ (pH 7.67), 3 M LiCl (pH 6.76), 1 M H_2_SO_4_ (pH 0.03), and 1 M KOH (pH 13.65) solutions. A piece of Celgard film was used as a separator (Celgard, USA). Two-electrode symmetric devices were assembled with two pieces of 0.2 cm^2^ TiN paper or pellet with the same area and mass loading (~ 1.5 mg cm^−2^). Three samples with similar mass loadings were tested for each condition to make sure the electrode’s capacitive performance is reproducible.

### Calculation

Gravimetric capacitance is calculated from CV curves using Eq. 1:

1 $$C_{\text{g}} = \frac{{\smallint I{\text{d}}V}}{v\Delta Vm}$$

where *I* is the current (A), *V* is the working potential, *v* is the scan rate (V s^−1^), Δ*V* is the working voltage, *m* is the mass loading (g). $\smallint I{\text{d}}V$ corresponds to the area of the discharging parts. For the working potential in the positive region, it corresponds the area in the reductive part. For the working potential in the negative region, it corresponds to the area in the oxidation part.

Areal capacitance is calculated from CV curves using Eq. 2

2 $$C_{A} = \frac{{\smallint I{\text{d}}V}}{v\Delta VA}$$

where *I* is the current (A), *V* is the working potential, v is the scan rate (V s^−1^), Δ*V* is the working voltage, *A* is the working area (cm^2^). $\smallint I{\text{d}}V$ corresponds to the area of the discharging parts.

The characteristic time constant (*τ*_characteristic_) is calculated by Eq. 3:

3 $$\tau_{\text{characteristic}} = \frac{1}{{2\pi f_{\text{characteristic}} }}$$

where the *f*_characteristic_ is the frequency (Hz) at a phase degree of − 45° from the EIS measurement.

Imaginary capacitances (*C*”) were calculated by Eq. 4:

4 $$C^{{{\prime \prime }}} = \frac{{Z^{{\prime }} }}{{2\pi f\left| Z \right|^{2} }}$$

where *Z*′ (Ω) is the real part of *Z*, *Z* is the electrochemical impedance (Ω), *f* is the frequency (Hz) from the EIS measurement.

## Results and Discussions

**Fig. 1:**
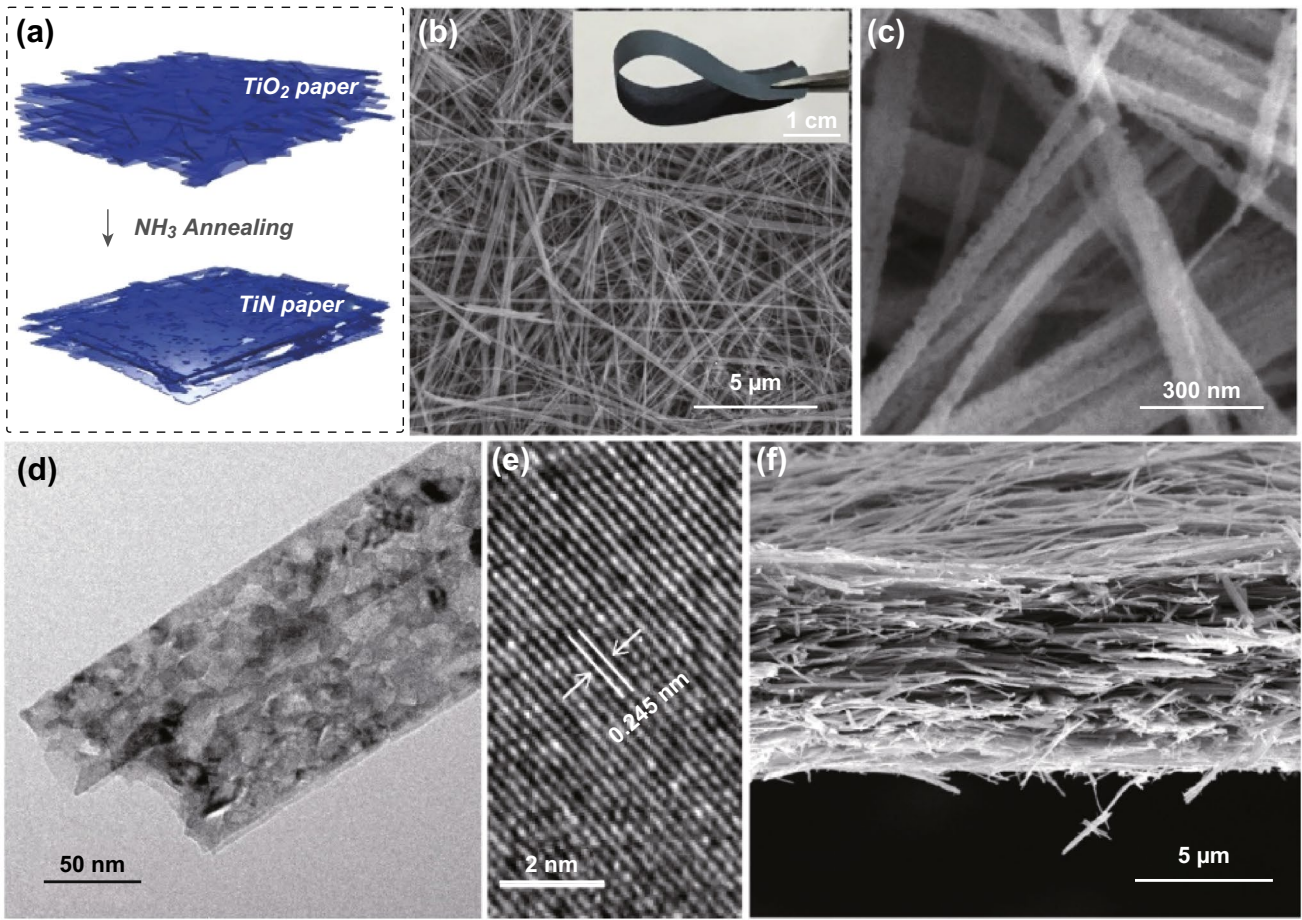
**a** Schematic illustration of the fabrication process of TiN paper from TiO_2_ paper. **b**, **c** SEM images of TiN nanobelts. Inset in **b** shows the digital image of a piece of flexible TiN paper. **d**, **e** TEM and high-resolution TEM images of porous TiN nanobelts. **f** Cross-sectional SEM image of TiN paper

Ultralong TiO_2_ nanobelts were prepared by a hydrothermal method. TiO_2_ nanobelts have average length around tens of micrometers, width of 50–200 nm and thickness of 20–50 nm (Fig. S1). These nanobelts with large aspect ratio can be easily assembled into flexible, paper-like electrodes using filtration method (Fig. S2) [26]. XRD patterns confirmed that the nanobelts are monoclinic TiO_2_ (TiO_2_-B, JCPDS No. 74-1940) (Fig. S3). TiN paper was obtained by treating the TiO_2_ paper in ammonia atmosphere at 800 °C (Fig. 1a). Notably, TiN paper inherits the excellent flexibility of TiO_2_ paper (Fig. 1b inset). While the basic framework of the 3D nanobelts assembly did not change upon ammonia treatment, each nanobelt became porous structure (Fig. 1b, c). N_2_ adsorption–desorption isotherms showed that the specific surface area increased from 31.2 m^2^ g^−1^ (TiO_2_ paper) to 43.5 m^2^ g^−1^ (TiN paper) after ammonia treatment (Fig. S4). The hysteresis located at 0.4 < P/P_0_ < 1.0 indicates the presence of mesopores [27]. The pore size distribution profile also confirmed the presence of higher amount of mesopores for TiN paper (Fig. S5). The porous structure was further confirmed with TEM image (Fig. 1d). It has been reported that TiO_2_ can be etched by ammonia at high temperatures [21, 22]. The topotactic reaction of TiO_2_ nanobelts and NH_3_ led to the rearrangement of the oxide structure and produced a mesoporous structure in the framework of the TiN nanobelts [28, 29]. The high-resolution TEM image revealed the TiN nanobelt has a lattice fringe of 0.245 nm, which is consistent with the *d*-spacing of (111) crystal plane of cubic TiN (Fig. 1e). The cross-sectional SEM image clearly showed that the TiN paper is formed via uniform assembly of nanobelts (Fig. 1f). The filtration method allows good control of the TiN paper thickness. The large pores between the nanobelts and mesoporous structure of the nanobelts provide sufficient space for ion diffusion, which is critical for the fast charging.

**Fig. 2:**
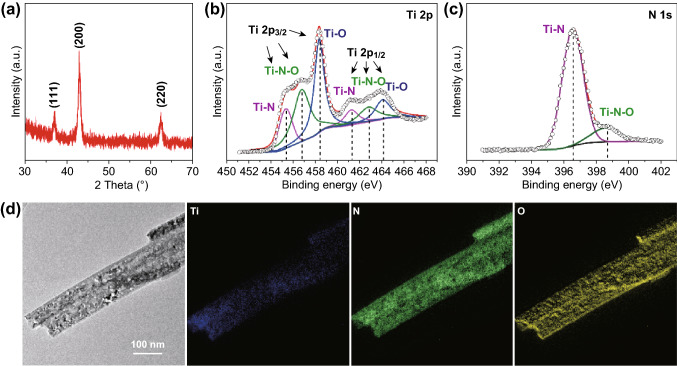
**a** XRD pattern and **b**, **c** Ti 2p and N 1s XPS spectra of TiN paper. **d** TEM image and the corresponding Ti, N, and O elemental mapping images of TiN nanobelts

Additional X-ray diffraction and spectroscopy techniques were used to probe the chemical composition of the TiN paper. Despite XRD pattern showed that the sample is cubic phase TiN (JCPDS No. 38-1420) (Fig. 2a), XPS survey spectrum revealed the existence of Ti, N, and O on the surface of TiN nanobelts (Fig. S6). The O signal is believed to be due to TiO_2_ and/or TiO_*x*_N_*y*_, which might come from the incomplete conversion from their oxide predecessor and surface oxidation after exposure in air [25, 30]. The possible reactions between TiO_2_ and NH_3_ are ammonia first decomposes into nitrogen and hydrogen gas ($2{\text{NH}}_{3} + {\text{heat}} \to {\text{N}}_{2} + 3{\text{H}}_{2}$). The reaction proceeds with the reduction of TiO_2_ to TiO by hydrogen gas (${\text{TiO}}_{2} + {\text{H}}_{2} \to {\text{TiO}} + {\text{H}}_{2} {\text{O}}$). Then, TiO reacts with ammonia and generate TiN (${\text{TiO}} + {\text{NH}}_{3} \to {\text{TiN}} + {\text{H}}_{2} {\text{O}}$) [31]. The TiO_2_ can also react with nitrogen and generate TiO_*x*_N_*y*_ (${\text{TiO}}_{2} + {\text{N}}_{2} \to {\text{TiO}}_{\text{x}}\, {\text{N}}_{\text{y}} + {\text{O}}_{2}$) [32].

The core-level Ti 2p XPS spectrum exhibits multiple peaks in the binding energy range between 453 and 466 eV. They can be deconvoluted into three sets of synthetic peaks, corresponding to Ti-N (2p_3/2_ = 455.4 eV, 2p_1/2_ = 461.3 eV), Ti-N–O (2p_3/2_ = 456.85 eV, 2p_1/2_ = 462.9 eV) and Ti–O (2p_3/2_ = 458.4 eV, 2p_1/2_ = 464.15 eV) (Fig. 2b) [33] The N 1s spectrum also consists of two different peaks, Ti-N (396.6 eV) and Ti-N–O (398.6 eV), which are consistent with the peaks observed in the Ti 2p spectrum (Fig. 2c) [34] Furthermore, TEM elemental mapping results confirmed the uniform distribution of Ti, N, and O in TiN nanobelts (Fig. 2d).

**Fig. 3:**
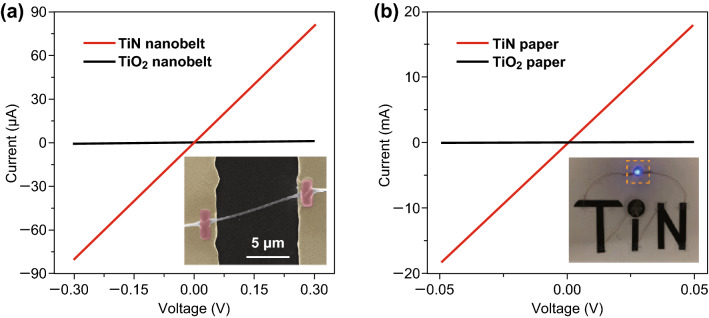
**a** *I*–*V* curves of individual ultralong TiO_2_ and TiN nanobelts. Inset shows the SEM image of a single nanobelt test unit. **b** *I*–*V* curves of TiO_2_ and TiN papers. Inset shows the digital image of TiN papers as a part of the electrical connections to light a blue LED by a commercial 3 V battery. (Color figure online)

To probe the electrical conductivity of TiO_2_ and TiN, focused ion beam (FIB) lithography was used to fabricate single-nanobelt devices (Fig. 3a inset). The current–voltage (*I*–*V*) curves of the TiO_2_ and TiN nanobelt devices are shown in Fig. 3a. TiN exhibits a significantly larger current response with voltage than TiO_2_. Their specific conductivities were calculated according to Eqs. 5 and 6:

5 $$R = \rho \frac{l}{A}$$

6 $$\sigma = \frac{1}{\rho }$$

where *R* is the resistance, *ρ* is the resistivity, *l* is the length of the nanobelt, *A* is the cross-sectional area of the nanobelt, and *σ* is the conductivity of the nanobelt. The conductivity of a single TiN nanobelt (4.5 × 10^5^ S m^−1^) is almost 3 orders of magnitude higher than that of TiO_2_ nanobelt (4.9 × 10^2^ S m^−1^). The TiN paper also retains excellent conductivity of 3.67 × 10^4^ S m^−1^. These values are much higher than the previous reported carbon-based materials, such as activated carbon (10–100 S m^−1^) [35], chemical converted graphene (5 × 10^2^ S m^−1^) [20], holy graphene (~ 10^3^ S m^−1^) [36], laser-scribed graphene (1738 S m^−1^) and even higher than the commercial CNT (~ 10^4^ S m^−1^) [37].

Furthermore, the TiN paper has a low sheet resistance of only 2.73 Ω sq^−1^, which is smaller than those of graphene film (280 Ω sq^−1^) [38], chemical converted graphene film (124 Ω sq^−1^) [39], CNT paper (10 Ω sq^−1^) [40], Au paper (7 Ω sq^−1^) [41], MoO_3−*x*_ paper (5.1 Ω sq^−1^) [37], Polypyrrole paper (4.5 Ω sq^−1^) [9], and comparable to that for poly(3,4-ethylenedioxythiophene):polystyrene sulfonate (PEDOT:PSS) paper (2.60 Ω sq^−1^) [42] (Fig. 3b). Given the excellent conductivity, the TiN paper can actually be used as a connecting lead to power a 2.5 V light-emitting-diode (LED) with a commercial 3 V button battery (Figs. 3b inset and S7).

The unique combination of high conductivity and pore structure makes TiN paper an excellent electrode candidate for ultrafast charging supercapacitors. The working potential of TiN papers was first evaluated using a three-electrode system in electrolytes with different pH values. TiN paper electrodes exhibit the largest working potential window (1.5 V) in 0.5 M Na_2_SO_4_ (pH 7.57) neutral electrolyte, while only 0.8 V in 1 M KOH (pH 13.65), 1 V in 1 M H_2_SO_4_ (pH 0.03), and 0.9 V in 3 M LiCl (pH 6.76) (Fig. S8). Significantly, this is the first report for TiN-based materials to be operated in such a wide working voltage of 1.5 V [22, 33, 43–46]. The large working voltage can be ascribed to the employment of the sulfate-based neutral electrolyte, which have been demonstrated to be effective in expanding the working voltage of SC materials in aqueous electrolyte because the high solvation energy of sulfate and alkali metal ions (160–220 kJ mol^−1^) cause relatively large overpotentials for hydrogen evolution and oxygen evolution reactions [47–50].

Excellent specific capacitances have been obtained for electrodes with small mass loading (0.1–1 mg cm^−2^) of metal nitrides deposited on conducting substrates (~ 10 to 200 mg cm^−2^) [21, 22, 24, 33, 51]. These capacitances were typically calculated based only on the mass of active material. However, the value of specific capacitance would be more practically meaningful if it is normalized to the mass of the entire electrode. In this regard, binder-free and conducting additive-free TiN papers are advantageous over its counterparts that require current collector. The TiN paper electrode showed a high capacitance of 164.5 F g^−1^ in 0.5 M Na_2_SO_4_ at a scan rate of 5 mV s^−1^ and retained 64.7% of its capacitance when the scan rate is raised to 100 mV s^−1^ (Fig. S9), which is significantly higher than capacitance of other metal nitride electrodes normalized to the mass of entire electrode, such as TiN nanosheets/graphene nanosheets (5.3 F g^−1^ at 10 mV s^−1^) [21], Nb_4_N_5_/Ni foil (0.86 F g^−1^ at 0.67 A g^−1^) [52], TiN nanowire/carbon cloth (10.2 F g^−1^ at 10 mV s^−1^) [22], VN nanowire/carbon cloth (16.7 F g^−1^ at 10 mV s^−1^) [53], and TiN/MnO_2_ nanowire/carbon cloth (25.9 F g^−1^ at 2 mA cm^−2^) [54].

**Fig. 4:**
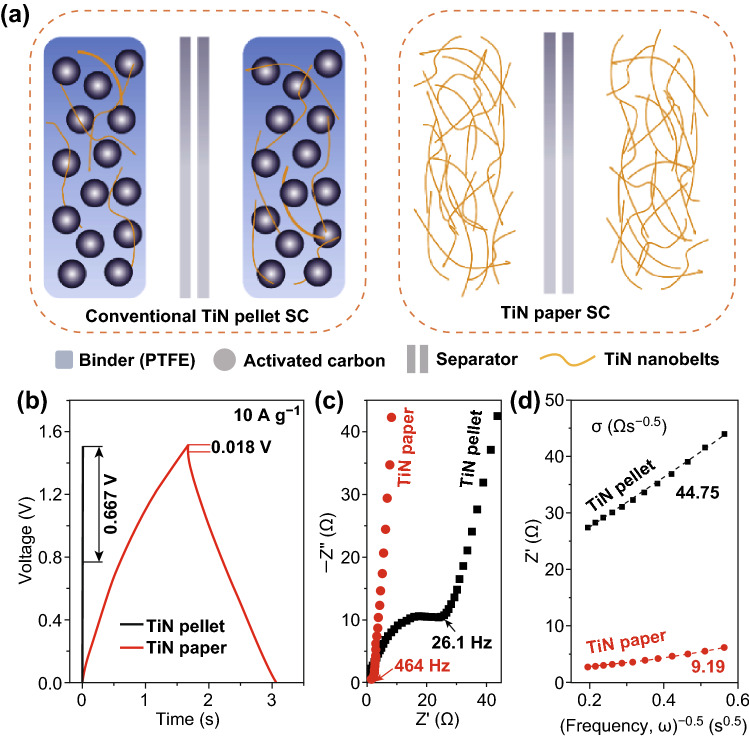
**a** Schematic illustration of the device configuration of TiN pellet and paper-based SSCs. **b** Galvanostatic charging and discharging curves of the SSCs at a current density of 10 A g^−1^. The iR drop of TiN paper SSC and pellet SSC are 0.018 and 0.667 V. **c** Electrochemical impedance spectra of TiN pellet SSC and TiN paper SSC. The knee frequency of the TiN paper SSCs and TiN pellet SSCs are 464 and 26.1 Hz. **d** *Z*′ versus the reciprocal of the square root of frequency (ω^−0.5^) in the intermediate frequency range. The dashed lines are the linear fitting lines for calculating the ion diffusion resistance, *σ*

TiN pellet electrodes as control samples were prepared by mixing TiN nanobelts, carbon black and PTFE binders, followed by pressing the mixture into thin pellets (Figs. 4a and S10). Symmetric supercapacitors (SSCs) were prepared via the assembly of two TiN paper or pellet electrodes with the same mass loadings. As shown in Fig. 4b, the paper-based SSCs indeed have considerably longer charging and discharging time than the pellet SSCs. Importantly, the paper SSC exhibits excellent capacitive behavior even at ultrafast charging rates at 200 A g^−1^ (Fig. S11), while the charging rate of the pellet SSC is limited by its large internal resistances (Fig. S12). As shown in Fig. S13, the TiN paper-based SSC achieves a specific capacitance of 12.67 F g^−1^ at a scan rate of 100 mV s^−1^ and retains a capacitance of 8.99 F g^−1^ at a high scan rate of 1 V s^−1^ and 3.35 F g^−1^ at an ultrahigh scan rate of 100 V s^−1^. These values are substantially higher than that of pellet-based SSC. The reduced capacitance and rate capability of the pellet electrode are mainly because of two reasons. First, the addition of non-conductive and non-electrochemical active polymer binder (PTFE) increases the overall electrode resistance and decreases the specific capacitance. Second, part of the active capacitive material TiN nanobelts is covered by carbon black and PTFE. This makes the ion diffusion to TiN nanobelts more difficult, especially at high charging rates, compared to the porous paper electrode.

EIS measurements were performed to understand the electron transport, ion diffusion resistivity and frequency characteristics of TiN SSCs. The equivalent series resistance (ESR) obtained from the intercept of the plot on the real axis is only 0.92 Ω, indicating the excellent electrical conductivity and low resistance of TiN paper SSCs (Fig. S14). Besides, the paper SSC showed much smaller charge transfer resistance (1.66 Ω) than the pellet SSC (35.98 Ω) (Fig. 4c). The high knee frequency of paper SSC (464 Hz) is also an order of magnitude higher than the knee frequency of the pellet SSC (26.1 Hz) (Fig. 4c). The characteristic time constant of the paper SSC was calculated to be 4 ms (Fig. S15). This value is substantially smaller than the values reported for many carbon-based SSCs, including CNT fibers (1930 ms) [55], metal–organic frameworks derived porous carbon (1270 ms) [56], carbide-derived carbon (379 ms) [57], laser-scribed graphene (33 ms) [58], and onion-like carbon (26 ms) [59].

In addition to the efficient electron transport, paper SSCs exhibited much faster ion diffusion kinetics than the conventional pellet SSCs. The ion diffusion resistances (*σ*) can be extracted from the slopes of the linear fitting of the real part of impedance (*Z*’) versus the reciprocal of the square root of frequency (ω^−0.5^) in the intermediate frequency range [60]. The paper SSC displayed an *σ* of 9.19 Ω s^−0.5^, which is much smaller than the pellet SSCs (44.75 Ω s^−0.5^), highlighting the advantage of having the unique porous electrode structure (Fig. 4d).

**Fig. 5:**
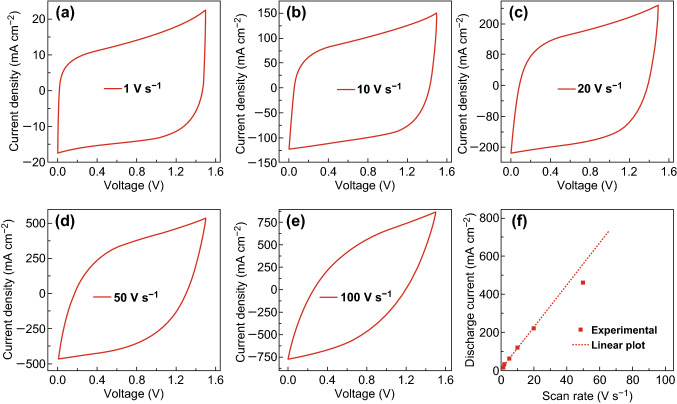
**a**–**e** CV curves of TiN paper SSC collected at different scan rates in 0.5 M Na_2_SO_4_ aqueous electrolyte. **f** A plot of discharge current of TiN paper SSC against scan rate

The CV curves of the paper SSC retain the rectangular shape even at ultrafast charging rates of 100 V s^−1^ (Fig. 5a–e). The linear increase of the discharge currents to 20 V s^−1^ reflects the efficient charge transfer and ion diffusion in the paper SSCs (Fig. 5f), in contrast to the pellet SSCs. The paper SCC delivers an energy density of 1.05 Wh kg^−1^ under an extraordinarily high power density of 251.2 kW kg^−1^, with a charging/discharging time of only 15 ms. These values are much better than most of previously reported electrochemical capacitors (Fig. S16). Furthermore, the TiN paper SSCs showed remarkable energy density of 3.26 mWh cm^−3^ under a power density of 78.3 mW cm^−3^. An energy density of 0.54 mWh cm^−3^ was still retained under an extremely high power density of 130,632.2 mW cm^−3^, which is again much higher than most metal nitride-based SSCs (Fig. S17) [21, 22, 51, 53, 61, 62].

**Fig. 6:**
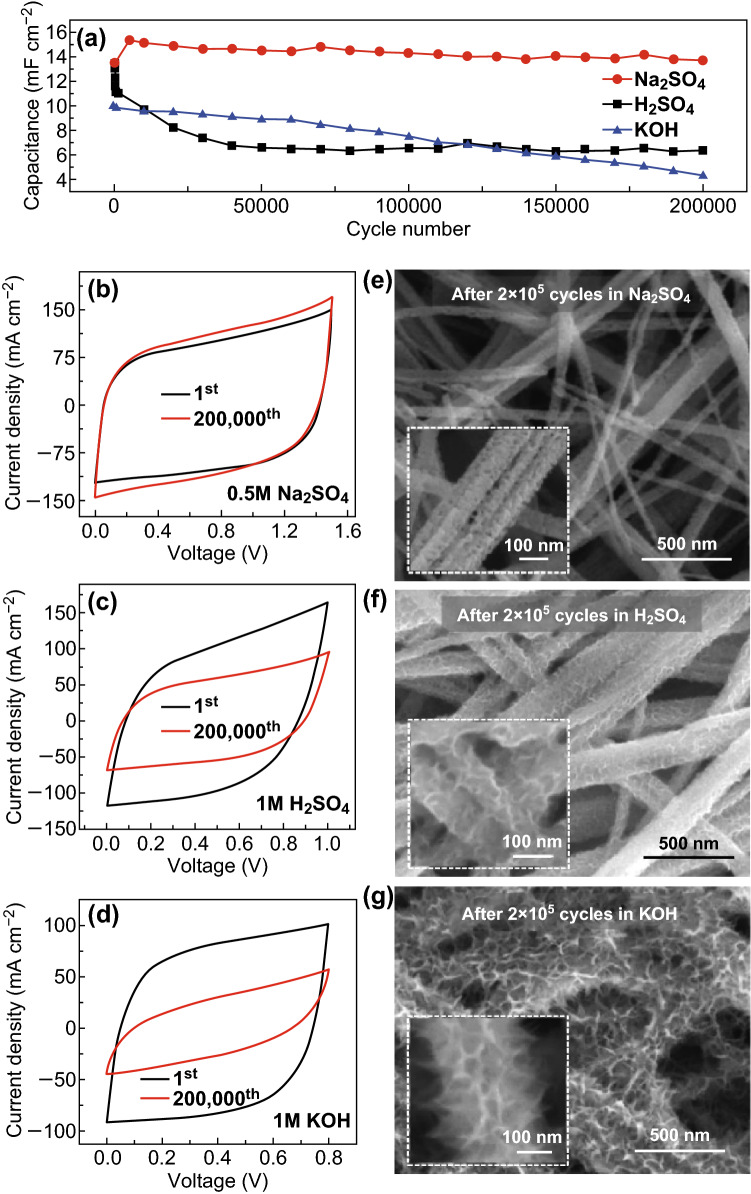
**a** Capacitance retention of TiN paper SSC obtained at 1 V s^−1^ in 0.5 M Na_2_SO_4_, 1 M H_2_SO_4_, and 1 M KOH electrolytes are plotted as a function of cycling number. **b**–**d** CV curves of TiN paper SSC collected at the 1st and after the 200,000th cycle in 0.5 M Na_2_SO_4_, 1 M H_2_SO_4_, and 1 M KOH electrolytes at 10 V s^−1^. **e**–**g** SEM images of the TiN paper electrodes after testing for 200,000 cycles in 0.5 M Na_2_SO_4_, 1 M H_2_SO_4_, and 1 M KOH electrolytes at 1 V s^−1^

Transition metal nitride electrodes have been suffering from the instability problem during cycling [22, 24]. TiN paper SSCs were tested for long-term stability in three different electrolytes, 0.5 M Na_2_SO_4_, 1 M H_2_SO_4_, and 1 M KOH solutions. TiN paper SSC shows zero decay in capacitance after cycling in 0.5 M Na_2_SO_4_ electrolyte for 200,000 cycles at 1 V s^−1^, while only 47.5% and 42.4% of capacitance were retained in 1 M H_2_SO_4_ and 1 M KOH electrolyte, respectively (Fig. 6a–d). SEM images revealed that the porous structure of TiN nanobelts remained unchanged after cycling in 0.5 M Na_2_SO_4_ electrolyte. In contrast, the nanobelt morphology of TiN changed significantly after testing in 1 M H_2_SO_4_ and 1 M KOH solutions (Fig. 6e–g). XPS spectra were collected to investigate the chemical nature of TiN electrode surface before and after cycling stability test. The N 1s spectrum of TiN paper tested in 0.5 M Na_2_SO_4_ solution shows no obvious change, while the N 1s peaks disappeared after cycling in 1 M H_2_SO_4_ or 1 M KOH electrolytes (Fig. S18). Ti 2p XPS spectra further showed that the signal of Ti-N and Ti-N–O decreased considerably after testing in H_2_SO_4_ or KOH solution, leaving only Ti–O signals, while the Ti-N and Ti-N–O signals were not affected for TiN paper tested in 0.5 M Na_2_SO_4_. These results suggested that TiN papers were oxidized in both H_2_SO_4_ and KOH solutions, which are consistent with the previous reports [25, 63, 64]. Titanium ions in TiN can be oxidized to soluble titanate ions (HTiO_3_^−^) and/or TiO_2_·H_2_O in alkaline solution [63], while they can also be oxidized steadily to the trivalent state (Ti(OH)^2+^) and tetravalent state (Ti(OH)_2_^2+^) at positive voltages in acidic solutions [64]. These results demonstrate the importance of selecting the sulfate-based neutral electrolyte for TiN electrodes and possibly other nitride electrodes. The outstanding cycling stability of TiN with zero decay in capacitance after 200,000 cycles in 0.5 M Na_2_SO_4_ is much better than the previous reports on metal nitride electrodes (Table S1), such as TiN nanowires on carbon cloth (82% after 15,000 cycles) [22], Mo_2_N nanobelts/graphene (85.7% after 4000 cycles) [65], Nb_4_N_5_ nanobelts (80% after 1000 cycles) [51], VN/CNT composite (82% after 10,000 cycles) [61], and Fe_2_N/graphene (92.9% after 20,000 cycles) [21].

**Fig. 7:**
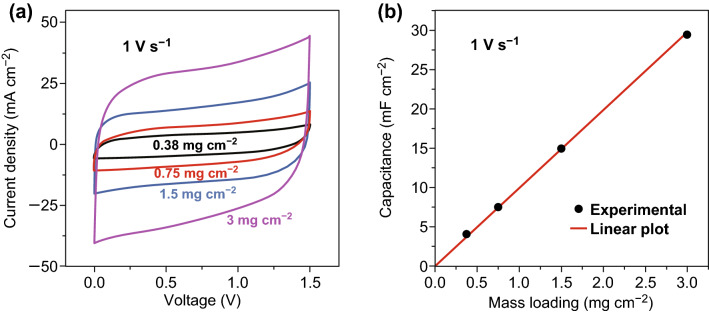
**a** CV curves of TiN paper SSC with different mass loadings collected at a scan rate of 1 V s^−1^. **b** Areal capacitance of TiN paper SSC is plotted as a function of TiN mass loading. The linear solid line is a guide to the eye

The filtration method offers not only an easy way to make paper-like electrode, but also the capability of controlling the electrode thickness and mass loading. We investigated the capacitive performance of TiN paper SSC with different mass loadings. As shown in Fig. 7a, TiN paper SSC retains a rectangular CV curves at a high scan rate of 1 V s^−1^ under different mass loadings. When the mass loading increased from 0.38 to 3 mg cm^−2^, the areal capacitance increases almost linearly, manifesting the gravimetric and volumetric capacitance are not significantly affected with the increased mass loading (Figs. 7b and S19). The thickness/mass loading-insensitive capacitive behavior makes the TiN paper electrode promising for practical energy storage devices.

## Conclusion

In summary, we have fabricated a freestanding highly conductive and porous TiN paper electrode that can be operated at an ultrahigh scan rate of 100 V s^−1^ in a wide voltage window of 1.5 V in a Na_2_SO_4_ electrolyte and shows no capacitance decay in 200,000 charge/discharge cycles. Importantly, the TiN paper SSC exhibits an outstanding response time with a characteristic time constant of 4 ms. This can be attributed to the high conductivity of TiN nanobelts and the efficient ion diffusion in the unique electrode architecture constructed with a network of mesoporous TiN nanobelts. We believe the paper-like electrode fabrication method can be applied to other metal nitride materials and provide an alternative way to make electrodes for ultrafast-charging supercapacitors.

## Electronic supplementary material

Below is the link to the electronic supplementary material.

Supplementary material 1 (PDF 1375 kb)
