# Supplementary material for: TiN Paper for Ultrafast-Charging Supercapacitors
Source: Nanomicro Lett. 2019 Dec 10;12:3. doi: 10.1007/s40820-019-0340-7 (PMC7770898; doi:10.1007/s40820-019-0340-7)
Supplement: Supplementary file 1 — Supplementary material 1 (PDF 1375 kb) [file 40820_2019_340_MOESM1_ESM.pdf]

Supporting Information for

TiN Paper for Ultrafast-Charging Supercapacitors

Bin Yao[1], Mingyang Li[1, 2], Jing Zhang[1], Lei Zhang[1, 3], Yu Song[1], Wang Xiao[1], Andrea Cruz[1], Yexiang Tong[2], Yat Li[1, *]

[1]Department of Chemistry and Biochemistry, University of California, Santa Cruz, California 95064, United States

[2]KLGHEI of Environment and Energy Chemistry, MOE of the Key Laboratory of Bioinorganic and Synthetic Chemistry, MOE of the Key Laboratory of Bioinorganic and Synthetic Chemistry, School of Chemistry and Chemical Engineering, Sun Yat-Sen University, Guangzhou 510275, People's Republic of China

[3]State Key Laboratory of Advanced Technology for Materials Synthesis and Processing, Wuhan University of Technology, Wuhan 430070, People's Republic of China

*Corresponding author. E-mail: yatli@ucsc.edu (Yat Li)

Supplementary Figures

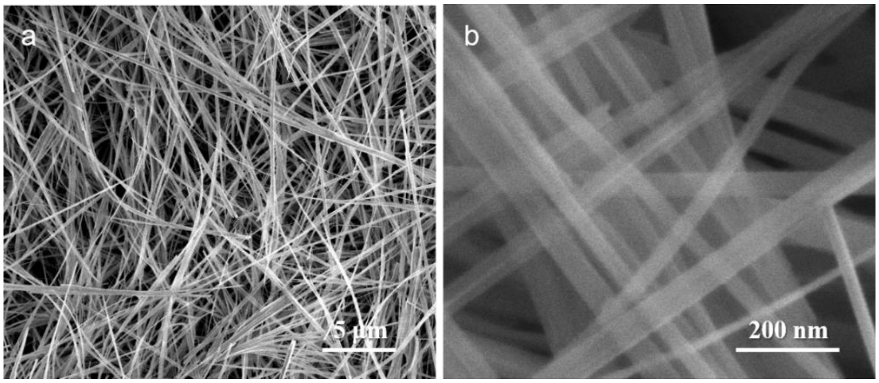

Fig. S1 SEM images of TiO_2 paper

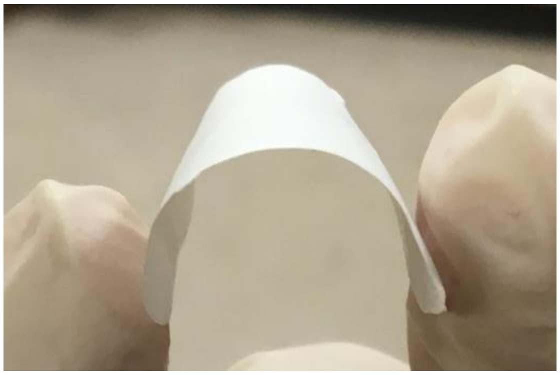

Fig. S2 Optical image of a piece of flexible TiO_2 paper

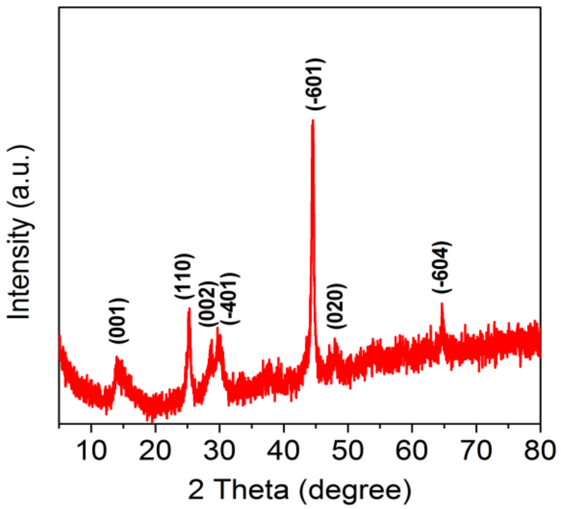

Fig. S3 XRD pattern of a TiO_2 paper. The diffraction peaks are consistent with the values reported for monoclinic phase TiO_2 (TiO_2-B) (JCPDS No. 74-1940)

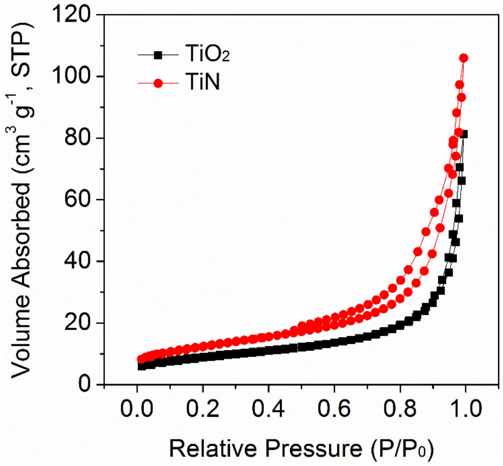

Fig. S4 N_2 adsorption/desorption isotherms of TiO_2 nanobelts and TiN nanobelts

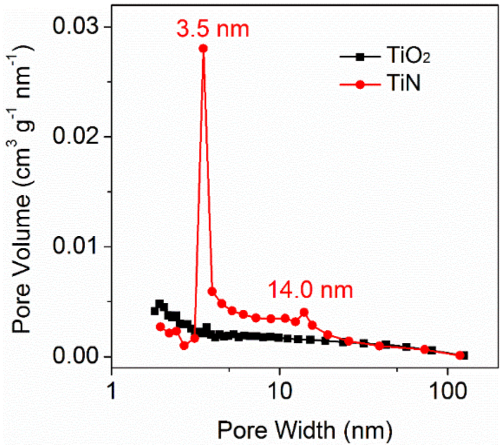

Fig. S5 Pore size distribution of TiO_2 nanobelts and TiN nanobelts

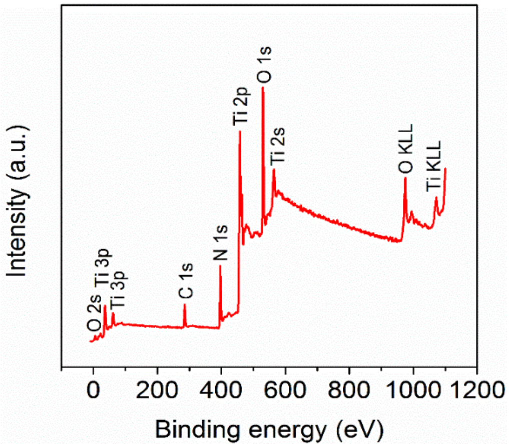

Fig. S6 XPS survey spectrum of TiN nanobelts

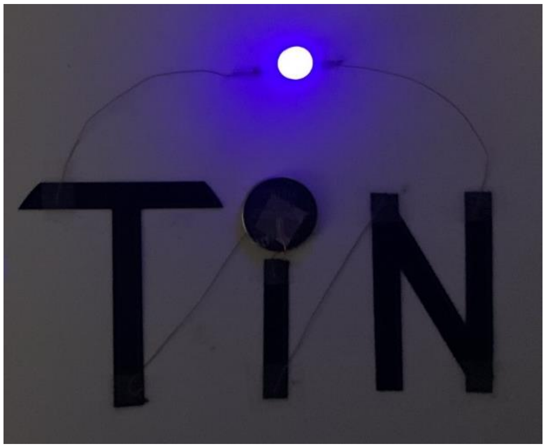

Fig. S7 A digital image of TiN papers as a part of the electrical connections to light a blue LED by a commercial 3V battery in dark environment

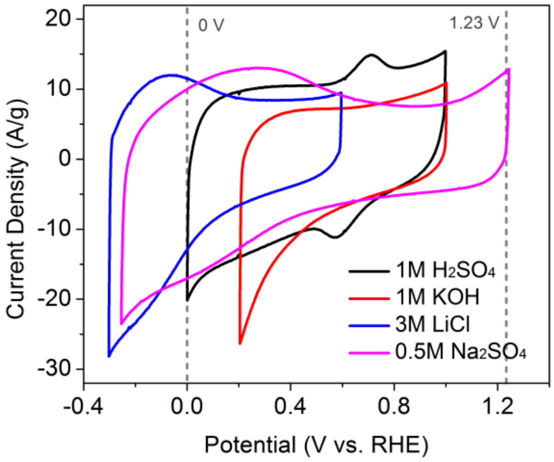

Fig. S8 CV curves of TiN paper electrodes obtained in electrolytes (1 M H_2SO_4, 1 M KOH, 3 M LiCl, and 0.5 M Na_2SO_4) with different pH values

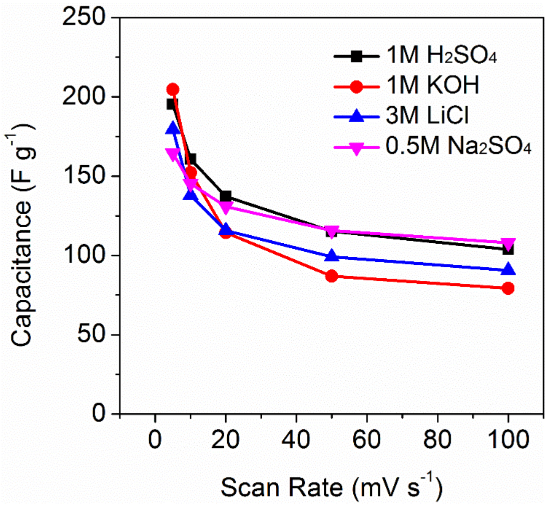

Fig. S9 The capacitance of TiN paper electrodes obtained in different electrolytes are plotted as a function of scan rate

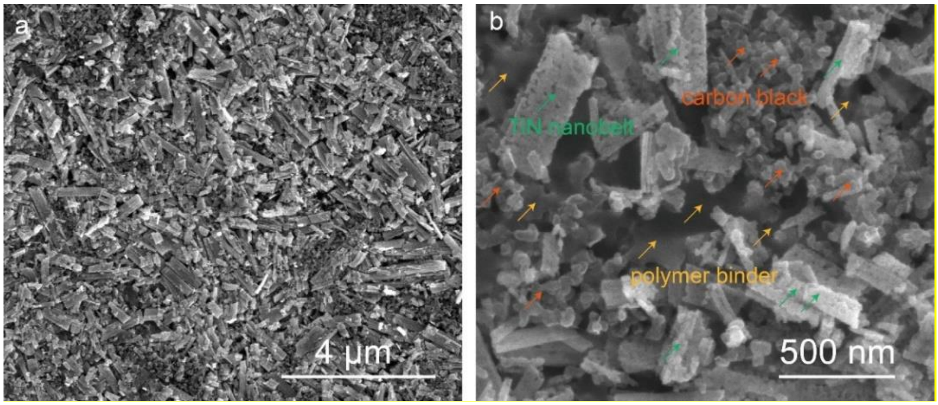

Fig. S10 SEM images of TiN pellet electrodes

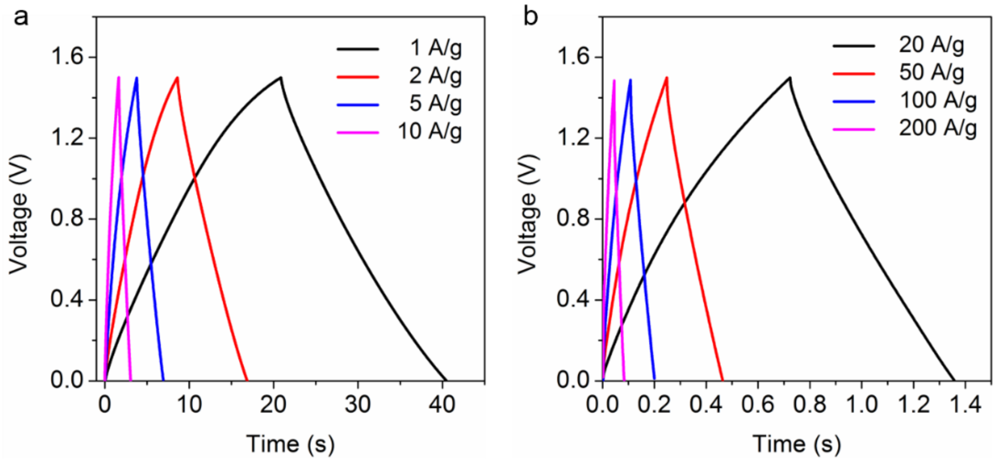

Fig. S11 Galvanostatic charging and discharging curves of TiN paper SSC obtained at different current densities

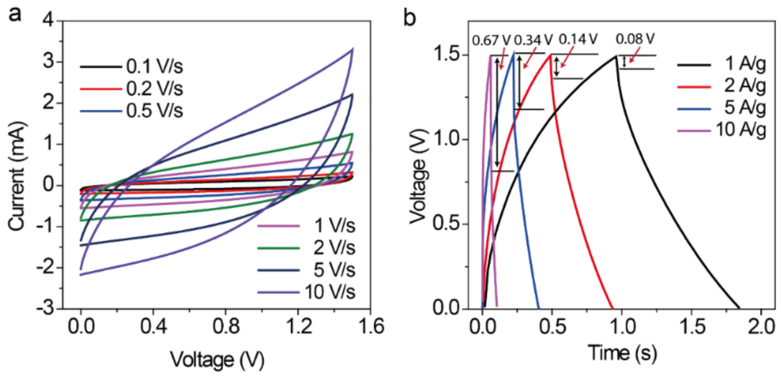

Fig. S12 a CV curves and **b** galvanostatic charging and discharging curves of conventional TiN pellet SSC

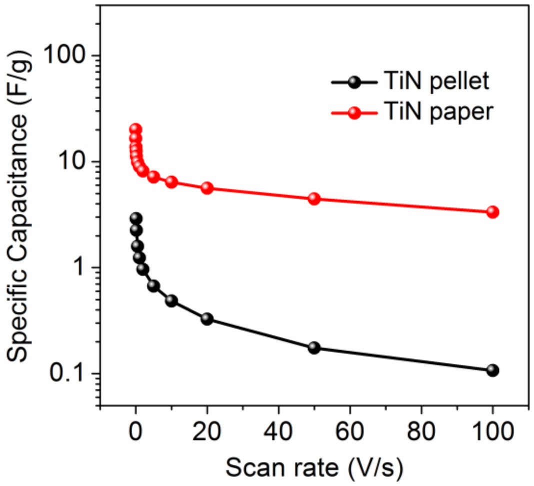

Fig. S13 Plots of specific capacitance of TiN pellet SSC and TiN paper SSC as a function of scan rate

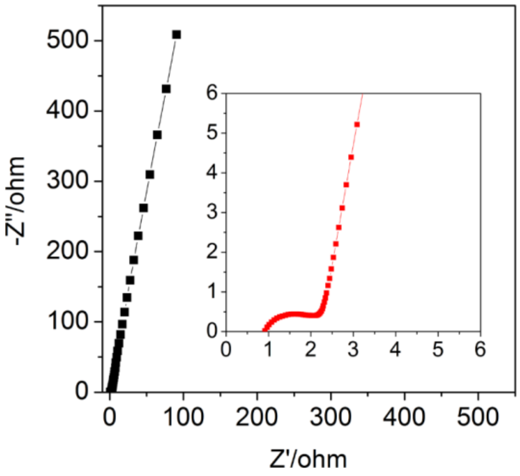

Fig. S14 Electrochemical impedance spectrum of TiN paper SSC. Inset shows the enlarged EIS spectrum at high frequencies

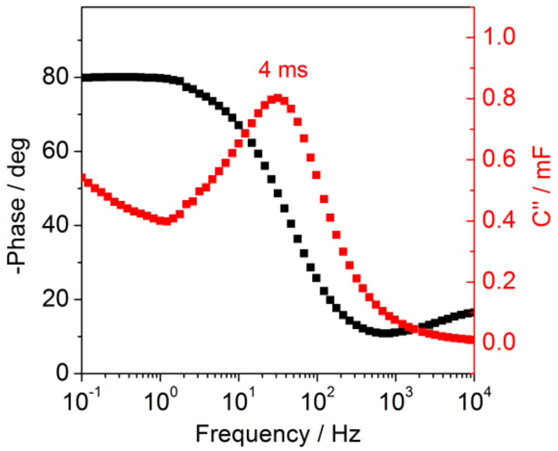

Fig. S15 Bode phase plot and imaginary capacitance (C″) of TiN paper electrode obtained at different frequencies

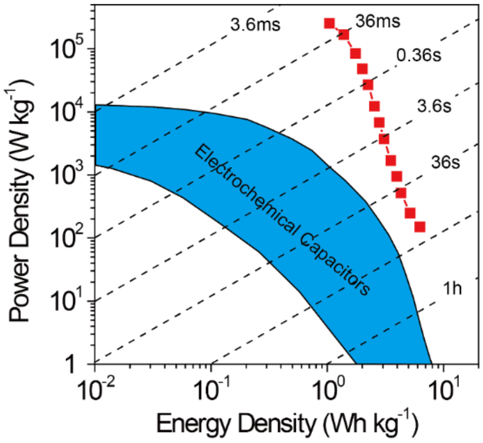

Fig. S16 A plot compares the energy density and power density of TiN paper SSCs and the previous SCs

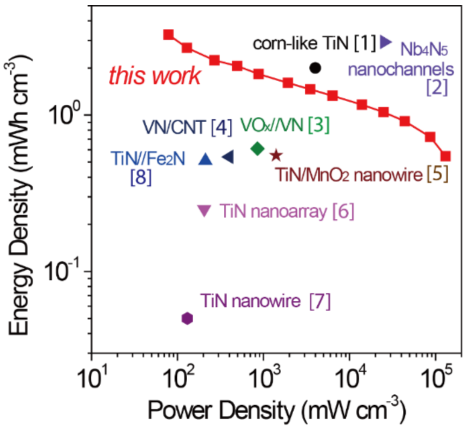

Fig. S17 A plot compares the energy density and power density of TiN paper SSCs and other metal nitride based SCs (corn-like TiN,[S1] Nb_4N_5 nanochannels,[S2] VO_x//VN,[S3] VN/CNT,[S4] TiN/MnO_2 nanowire,[S5] TiN nanoarray,[S6] TiN nanowire,[S7] TiN//Fe_2N[S8])

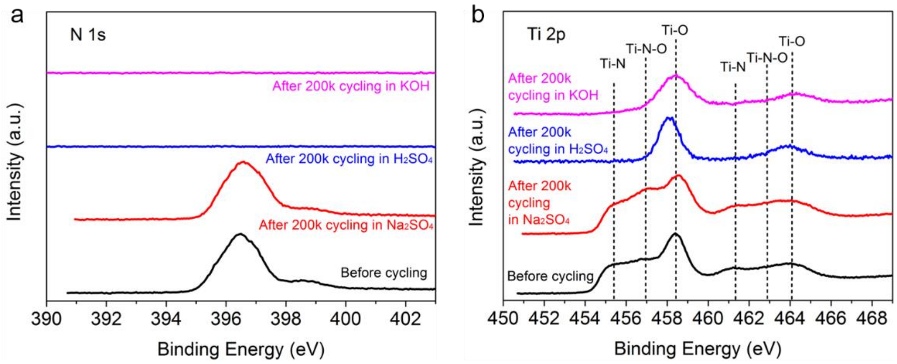

Fig. S18 Core level N 1s and Ti 2p XPS spectra collected for TiN paper electrodes after testing in different electrolytes (0.5 M Na_2SO_4,1 M H_2SO_4 and 1 M KOH) for 200,000 cycles

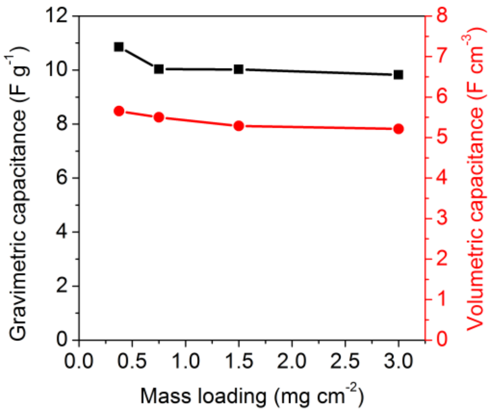

Fig. S19 Gravimetric capacitance and volumetric capacitance of TiN paper SSCs with different mass loadings of TiN at a high scan rate of 1 V s^{-1}

Table S1 Summary of the rate and cycling performance of transition metal nitride electrodes

**Materials**	**Highest scan rate**	**Cycling stability**	**Refs.**
TiN nanotubes	200 mV s^{-1}	98.5 % after 100 cycles at 2.5 mA cm^{-2}	[S6]
TiN nanowires	400 mV s^{-1}	82 % after 15000 cycles at 100 mV s^{-1}	[S7]
TiN/CNT	1 V s^{-1}	90 % after 20000 cycles at 100 mV s^{-1}	[S9]
PANI/TiN/PANI	200 mV s^{-1}	83 % after 3000 cycles at 0.2 mA cm^{-2}	[S10]
TiN/PPy	200 mV s^{-1}	72.6 % after 20000 cycles at 15 A g^{-1}	[S11]
TiN/$NiCo_2O_4$	200 mV s^{-1}	70 % after 20000 cycles at 10 mA cm^{-2}	[S12]
VN nanowires	100 mV s^{-1}	95.3 % after 10000 cycles at 100 mV s^{-1}	[S3]

VN nanobelts/NC	500 mV s^{-1}	91.8 % after 12000 cycles at 200 mV s^{-1}	[S13]
VN/CNT	100 mV s^{-1}	82 % after 10000 cycles at 0.2 A cm^{-3}	[S4]
VN/graphene	150 mV s^{-1}	94 % after 2000 cycles at 30 mV s^{-1}	[S14]
VN/$Co(OH)_2$	100 mV s^{-1}	86 % after 4000 cycles at 1 A g^{-1}	[S15]
Fe_2N/graphene	100 mV s^{-1}	92.9 % after 20000 cycles at 4 A g^{-1}	[S8]
GaN nanowires/graphene	100 V s^{-1}	98 % after 50000 cycles at 10 mA cm^{-2}	[S16]
Co_2N/Ni-doped Co	100 mV s^{-1}	82.5 % after 5000 cycles at 50 mV s^{-1}	[S17]
Nb_4N_5 nanobelts	1 V s^{-1}	80 % after 1000 cycles	[S18]
Nb_4N_5 nanochannels	200 mV s^{-1}	70.9 % after 2000 cycles at 50 mV s^{-1}	[S2]
MoN nanosheets	10 V s^{-1}	95 % after 25000 cycles at 100 mV s^{-1}	[S19]
Mo_2N nanobelts	200 mV s^{-1}	91 % after 1000 cycles at 100 mV s^{-1}	[S20]
Mo_2N nanobelts/graphene	1 V s^{-1}	85.7 % after 4000 cycles at 0.57 A cm^{-3}	[S21]
**TiN paper**	**100 V s^{-1}**	**102.2 % after 200,000 cycles at 1 V s^{-1}**	**this work**

Supplementary References

[S1] P. Yang, D. Chao, C. Zhu, X. Xia, Y. Zhang et al., Ultrafast-charging supercapacitors based on corn-like titanium nitride nanostructures. Adv. Sci. **3**, 1500299 (2016). http://doi.org/10.1002/advs.201500299

[S2] H. Cui, G. Zhu, X. Liu, F. Liu, Y. Xie et al., Niobium nitride Nb_4N_5 as a new high-performance electrode material for supercapacitors. Adv. Sci. **2**, 1500126 (2015). http://doi.org/10.1002/advs.201500126

[S3] X. Lu, M. Yu, T. Zhai, G. Wang, S. Xie et al., High energy density asymmetric quasi-solid-state supercapacitor based on porous vanadium nitride nanowire anode. Nano Lett. **13**, 2628-2633 (2013). http://doi.org/10.1021/nl400760a

[S4] X. Xiao, X. Peng, H. Jin, T. Li, C. Zhang et al., Freestanding mesoporous VN/CNT hybrid electrodes for flexible all-solid-state supercapacitors. Adv. Mater. **25**, 5091-5097 (2013). http://doi.org/10.1002/adma.201301465

[S5] Y. Liu, R. Xiao, Y. Qiu, Y. Fang, P. Zhang, Flexible advanced asymmetric supercapacitors based on titanium nitride-based nanowire electrodes. Electrochim. Acta **213**, 393-399 (2016). http://doi.org/10.1016/j.electacta.2016.06.166

[S6] Y. Xie, Y. Wang, H. Du, Electrochemical capacitance performance of titanium nitride nanoarray. Mater. Sci. Eng. B **178**, 1443-1451 (2013). http://doi.org/10.1016/j.mseb.2013.09.005

[S7] X. Lu, G. Wang, T. Zhai, M. Yu, S. Xie et al., Stabilized TiN nanowire arrays for high-performance and flexible supercapacitors. Nano Lett. **12**, 5376-5381 (2012). http://doi.org/10.1021/nl302761z

[S8] C. Zhu, P. Yang, D. Chao, X. Wang, X. Zhang et al., All metal nitrides solid-state asymmetric supercapacitors. Adv. Mater. **27**, 4566-4571 (2015). http://doi.org/10.1002/adma.201501838

[S9] A. Achour, J.B. Ducros, R.L. Porto, M. Boujtita, E. Gautron et al., Hierarchical nanocomposite electrodes based on titanium nitride and carbon nanotubes for micro-supercapacitors. Nano Energy **7**, 104-113 (2014). http://doi.org/10.1016/j.nanoen.2014.04.008

[S10] X. Peng, K. Huo, J. Fu, X. Zhang, B. Gao, P. K. Chu, Coaxial PANI/TiN/PANI nanotube arrays for high-performance supercapacitor electrodes. Chem. Commun. **49**, 10172-10174 (2013). http://doi.org/10.1039/C3CC45997G

[S11] H. Du, Y. Xie, C. Xia, W. Wang, F. Tian, Electrochemical capacitance of polypyrrole–titanium nitride and polypyrrole–titania nanotube hybrids. New J. Chem. **38**, 1284-1293 (2014). http://doi.org/10.1039/C3NJ01286G

[S12] R.Q. Wang, C. Xia, N.N. Wei, H.N. Alshareef, $NiCo_2O_4$@TiN core-shell electrodes through conformal atomic layer deposition for all-solid-state supercapacitors. Electrochim. Acta **196**, 611-621 (2016). http://doi.org/10.1016/j.electacta.2016.03.015

[S13] B. Gao, X.X. Li, X.L. Guo, X.M. Zhang, X. Peng et al., Nitrogen-doped carbon encapsulated mesoporous vanadium nitride nanowires as self-supported electrodes for flexible all-solid-state supercapacitors. Adv. Mater. Interfaces **2**, 1500211-1500219 (2015). http://doi.org/10.1002/Admi.201500211

[S14] H.H. Liu, H.L. Zhang, H.B. Xu, T.P. Lou, Z.T. Sui, Y. Zhang, In situ self-sacrificed template synthesis of vanadium nitride/nitrogen-doped graphene nanocomposites for electrochemical capacitors. Nanoscale **10**, 5246-5253 (2018). http://doi.org/10.1039/c7nr08985f

[S15] R. Wang, X. Yan, J. Lang, Z. Zheng, P. Zhang, A hybrid supercapacitor based on flower-like $Co(OH)_2$ and urchin-like VN electrode materials. J. Mater. Chem. A **2**, 12724-12732 (2014). http://doi.org/10.1039/C4TA01296H

[S16] S. Wang, L. Zhang, C. Sun, Y. Shao, Y. Wu, J. Lv, X. Hao, Gallium nitride crystals: novel supercapacitor electrode materials. Adv. Mater. **28**, 3768-3776 (2016). http://doi.org/10.1002/adma.201600725

[S17] X. Liu, W. Zang, C. Guan, L. Zhang, Y. Qian et al., Ni-doped cobalt–cobalt nitride heterostructure arrays for high-power supercapacitors. ACS Energy Lett. **3**, 2462-2469 (2018). http://doi.org/10.1021/acsenergylett.8b01393

[S18] B. Gao, X. Xiao, J. Su, X. Zhang, X. Peng, J. Fu, P.K. Chu, Synthesis of mesoporous niobium nitride nanobelt arrays and their capacitive properties. Appl. Surf. Sci. **383**, 57-63 (2016). http://doi.org/10.1016/j.apsusc.2016.04.173

[S19] X. Xiao, H. Yu, H. Jin, M. Wu, Y. Fang et al., Salt-templated synthesis of 2D metallic MoN and other nitrides. ACS Nano **11**, 2180-2186 (2017). http://doi.org/10.1021/acsnano.6b08534

[S20] J. Liu, K. Huang, H.L. Tang, M. Lei, Porous and single-crystalline-like molybdenum nitride nanobelts as a non-noble electrocatalyst for alkaline fuel cells and electrode materials for supercapacitors. Int. J. Hydrog. Energy **41**, 996-1001 (2016). http://doi.org/10.1016/j.ijhydene.2015.11.086

[S21] G.Q. Ma, Z. Wang, B. Gao, T.P. Ding et al., Multilayered paper-like electrodes composed of alternating stacked mesoporous Mo_2N nanobelts and reduced graphene oxide for flexible all-solid-state supercapacitors. J. Mater. Chem. A **3**, 14617-14624 (2015). http://doi.org/10.1039/c5ta02851e
